# Transcranial direct current stimulation combined with upper limb functional training in children with spastic, hemiparetic cerebral palsy: study protocol for a randomized controlled trial

**DOI:** 10.1186/s13063-016-1534-7

**Published:** 2016-08-17

**Authors:** Renata Calhes Franco Moura, Cibele Almeida Santos, Luanda André Collange Grecco, Roberta Delasta Lazzari, Arislander Jonathan Lopes Dumont, Natalia Carvalho de Almeida Duarte, Luiz Alfredo Braun, Jamile Benite Palma Lopes, Ligia Abram dos Santos, Eliane Lopes Souza Rodrigues, Giorgio Albertini, Veronica Cimolin, Manuela Galli, Claudia Santos Oliveira

**Affiliations:** 1 Rehabilitation Sciences, Nove de Julho University (UNINOVE), São Paulo, SP Brazil; 2 Hospital Servidor Publico, São Paulo, SP Brazil; 3 Mandaqui Hospital Complex, São Paulo, SP Brazil; 4 Motion Analysis Laboratory, IRCCS San Raffaele Pisana, Pisana, Rome Italy; 5 Department of Electronic Information and Bioengineering, Politecnico di Milano, Milan, Italy; 6 Rua Itapicuru, 380 apto 111, Perdizes bairro, CEP 05006-000 São Paulo, SP Brazil

**Keywords:** Cerebral palsy, Electrical stimulation, Physical therapy

## Abstract

**Background:**

The aim of the proposed study is to perform a comparative analysis of functional training effects for the paretic upper limb with and without transcranial direct current stimulation over the primary motor cortex in children with spastic hemiparetic cerebral palsy.

**Methods:**

The sample will comprise 34 individuals with spastic hemiparetic cerebral palsy, 6 to 16 years old, classified at level I, II, or III of the Manual Ability Classification System. Participants will be randomly allocated to two groups: (1) functional training of the paretic upper limb combined with anodic transcranial stimulation; (2) functional training of the paretic upper limb combined with sham transcranial stimulation. Evaluation will involve three-dimensional movement analysis and electromyography using the SMART-D 140® system (BTS Engineering) and the FREEEMG® system (BTS Engineering), the Quality of Upper Extremity Skills Test, to assess functional mobility, the Portable Device and Ashworth Scale, to measure movement resistance and spasticity, and the Pediatric Evaluation of Disability Inventory, to evaluate performance. Functional reach training of the paretic upper limb will include a range of manual activities using educational toys associated with an induced constraint of the non-paretic limb during the training. Training will be performed in five weekly 20-minute sessions for two weeks. Transcranial stimulation over the primary motor cortex will be performed during the training sessions at an intensity of 1 mA. Findings will be analyzed statistically considering a 5 % significance level (*P* ≤ 0.05).

**Discussion:**

This paper presents a detailed description of a prospective, randomized, controlled, double-blind, clinical trial designed to demonstrate the effects of combining transcranial direct current stimulation over the primary motor cortex and functional training of the paretic limb in children with cerebral palsy classified at level I, II, or III of the Manual Ability Classification System. The results will be published and evidence found may contribute to the use of transcranial stimulation for this population.

**Trial registration:**

ReBEC RBR-6V4Y3K. Registered on 11 February 2015.

## Background

Cerebral palsy is a term used for motor development disorders stemming from a primary brain lesion that are permanent and mutable, causing secondary musculoskeletal problems and limitations regarding activities of daily living [1]. Motor impairment is the main manifestation of cerebral palsy, leading to abnormal body biomechanics. Children with cerebral palsy may also exhibit intellectual and sensorial impairments, which further restrict functional performance [2, 3].

Depending on the topographic distribution, motor impairment is categorized as tetraplegia when all limbs are affected, which accounts for 9–43 % of cases, diplegia when upper limb involvement is milder than lower limb involvement, which accounts for 10–33 % of cases, and hemiplegia when only one side of the body is affected, which accounts for 25–40 % of cases. The upper limbs are affected in 50–70 % of individuals with cerebral palsy [4, 5], with a highly variable dynamic upper limb movement pattern, depending on the location and extent of the central nervous system lesion. Hemiparesis is a milder form of hemiplegia, characterized by unilateral motor deficiency contralateral to the brain lesion [6, 7]. Together with muscle spasticity, children with hemiparesis exhibit loss of motor neuronal excitation, which is typically associated with poor selective motor control and muscle weakness, resulting in significant functional incapacity [6, 8, 9].

Children with hemiparesis have limitations regarding the use of the affected upper limb and, consequently, two-hand coordination; this exerts a negative impact on activities of daily living and participation at school, in the community, and in family life [10]. Spasticity, muscle weakness, limited supination, and limited reach lead to difficulties with activities involving reaching, grasping, and handling objects. Such problems compromise functional performance, especially among schoolchildren, who are required to demonstrate greater independence in activities related to learning, mobility, and self-care.

Primary upper and lower limb impairment due to spasticity, muscle weakness, and deficient motor control can give rise to secondary musculoskeletal complications, such as contractures and deformities, resulting in restricted movements. Functional limitations can result in motor deficiency, as well as impairments with regard to sensation, perception, cognition, behavior, and communication [4, 11]. According to Darrah et al. [12], functional performance in children with cerebral palsy is determined by a number of factors, such as abnormal muscle tone, the demands of a given task, the severity of the clinical condition, and aspects of the environment. Moreover, motivation, interest, family support, proper adaptations, and opportunities to practice a given task have a positive influence on functional performance in these children. Considering these factors, many therapies are directed toward improving neuromuscular deficiencies with the intention of improving functional performance [4, 13, 14]. Different intensive therapy approaches are currently aimed at improving the performance of the upper limbs. Traditional therapy uses a two-hand training approach; whereas constraint-induced movement therapy has emerged as a promising single-hand training approach [10]. However, a meta-analysis evaluating the efficacy of all non-surgical methods for the upper limb rehabilitation stresses the absence of strong evidence supporting a specific therapy model to improve the functional performance of the affected upper limb in children with hemiparesis secondary to cerebral palsy [15].

### Transcranial direct current stimulation

Transcranial direct current stimulation is a promising non-invasive therapeutic resource for the treatment of children with cerebral palsy in which the motor cortex is stimulated using a low-intensity (1–2 mA), monophasic, direct, electrical current through surface electrodes. The advantages of transcranial direct current stimulation over other transcranial stimulation methods are the longer-lasting modulating effect on cortex function, ease of administration, and lower cost. Moreover, this type of intervention allows better placebo stimulation, thereby conferring greater specificity to scientific findings [16, 17].

The effects of stimulation are achieved by the movement of electrons. The two electrodes are an anode with a positive charge and a cathode with a negative charge. An electrical current flows from the positive to the negative pole and has different effects on biological tissues. Although most of the current is dissipated among the tissues over the cortex during transcranial direct current stimulation, a sufficient amount reaches the cortex structures and modifies the membrane potential of local cells [18, 19].

Transcranial direct current stimulation has short-term effects on cortex excitability when administered for short periods and longer-lasting effects related to plastic mechanisms when administered over a longer period [20]. A number of studies conducted on animal models report the effects of transcranial direct current stimulation on the cerebral cortex, demonstrating that polarized currents administered to the cerebral surface increase spontaneous firing [21] and initiate paroxystic activity [22] when the anodal pole is used, whereas the cathode generally depresses these events. Based on these data, studies involving human beings have evaluated the effects of each pole on cortex excitability through stimulation of the primary motor cortex. Anodal stimulation is reported to increase excitability and cathodal stimulation diminishes excitability [21]. Transcranial direct current stimulation is a neuromodulation technique that has drawn the attention of a large number of researchers in recent years. The findings of clinical studies have demonstrated the potential of this method in the treatment of neurological disorders and the investigation of the modulation of cortex excitability [17].

In the rehabilitation process, the aim of neuromodulation techniques is to increase local synaptic efficacy, thereby altering the maladaptive plasticity pattern that emerges following a cortex lesion. The possibility of combining physical therapy modalities is one of the advantages of transcranial direct current stimulation. Stimulation is a way to modulate cortex activity by opening a path to enhance and prolong functional gains achieved through physical therapy. Stimulation allows a change in a dysfunctional excitability pattern through the activation of specific neural networks so that physical therapy can mold a functional cortex activity pattern [17]. Studies involving the use of transcranial direct current stimulation over the primary motor cortex in stroke victims have demonstrated improvements in upper limb function (active wrist and finger movements), movement velocity, active ankle movements, and overall motor function. However, only a very small number of studies have analyzed the effects of transcranial stimulation in children with cerebral palsy. The literature reports the use of transcranial magnetic stimulation as a way to analyze evoked potentials [23, 24] and as a resource to reduce spasticity in children with cerebral palsy [25, 26].

Analyzing the effects of transcranial magnetic stimulation, a recent study [27] reports significant changes in motor cortex maps in children with hemiparesis or diparesis stemming from cerebral palsy, such as lateral movements of the affected upper limb and motor representation of the affected lower limb, demonstrating reorganization following lesions in one or both hemispheres of the brain. Another recent study [28] employed transcranial direct current stimulation combined with treadmill training in a child with cerebral palsy and found beneficial effects with regard to the acquisition of motor skills. Another study [29], involving children with congenital hemiparesis, concluded that transcranial direct current stimulation appears to be safe, feasible, and well tolerated in most children with hemiparesis, but the authors of that study suggest further investigations of serial sessions of transcranial direct current stimulation in conjunction with rehabilitation for a synergistic approach to improving hand function.

## Methods/design

### Primary objective

The primary objective of the proposed study is to analyze manual ability before and after functional training of the paretic upper limb with anodic and sham transcranial direct current stimulation over the primary motor cortex (ipsilesional hemisphere) in children with spastic, hemiparetic cerebral palsy, classified at levels I, II, or III of the Manual Ability Classification System [30].

### Hypothesis 1

Functional reach training of the paretic upper limb with anodic transcranial direct current stimulation over the primary motor cortex will achieve greater effects in comparison to functional reach training with sham transcranial direct current stimulation in children with spastic, hemiparetic cerebral palsy classified at level I, II, or III of the Manual Ability Classification System [30].

### Study design

**Fig. 1:**
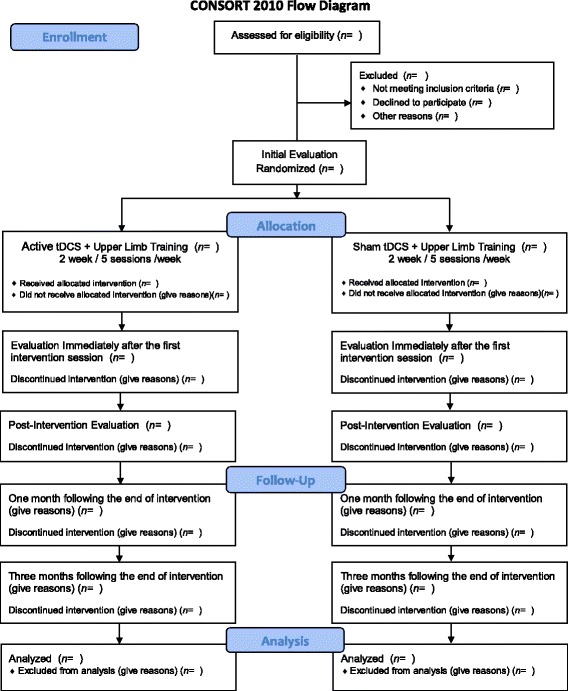
Flowchart of the study following Consolidated Standards of Reporting Trials (CONSORT) guidelines. tDCS, transcranial direct current stimulation

A prospective, paired, randomized, controlled, double-blind clinical trial is proposed (Fig. 1). The protocol for this study is registered with the Brazilian Registry of Clinical Trials (ReBEC) RBR-6V4Y3K.

### Recruitment and sample selection

Individuals with hemiparetic cerebral palsy will be recruited from the physical therapy clinics of the Nove de Julho University, São Paulo, Brazil, and will be selected based on the following eligibility criteria.

### Inclusion criteria

- Diagnosis of spastic, hemiparetic cerebral palsy
- Functional classification at level I, II, or III of the Manual Ability Classification System [30]
- Aged 6 to 16 years
- Adequate understanding and cooperation during the proposed activities
- Statement of informed consent signed by a legal guardian

### Exclusion criteria

- Having undergone surgical procedures or neurolytic block in the 12 months prior to the onset of the training sessions
- Orthopedic deformity with indication for surgery
- Epilepsy
- Metal implant in the skull or use of hearing aids

### Sample size

The sample size was calculated using the STATA 11 program, using a study conducted by Choudhary et al. [31] as the basis. Scores on the Quality of Upper Extremity Skills Test (QUEST) were used for the calculation. Based on the mean and standard deviation values of the experimental group prior to intervention (mean: 76.4; standard deviation: 9.2) and after the intervention (mean: 87.2; standard deviation: 9.4), a bidirectional alpha of 0.05 and 80 % test power, a minimum of 13 children were deemed necessary for each group. The sample will be increased by 20 % to compensate for possible dropouts, leading to 17 children in each group (overall sample: 34 participants).

### Randomization

Children who meet the eligibility criteria will be randomly allocated to one of the two study groups using block randomization.

### Group 1

This group will receive the intervention, that is, functional reach training of the paretic upper limb with range manual activities combined with active transcranial direct current stimulation over the primary motor cortex of the ipsilesional hemisphere.

### Group 2

This will be the ‘control’ group and will receive functional reach training of the paretic upper limb with range manual activities combined with sham transcranial direct current stimulation over the primary motor cortex of the ipsilesional hemisphere.

### Allocation concealment

The children will be randomly allocated to the two groups. To minimize the risk of an imbalance in the size of the groups, a randomization list will be generated using five blocks of six participants, with three participants in each block randomly allocated to each group, and one block of four participants, with two participants in each block randomly allocated to each group. The allocation sequence will be stipulated in sequentially numbered, sealed, opaque envelopes. Following the baseline evaluation, each participant will be allocated to one of the groups by opening an envelope. This process will be performed by a member of the research team who is not involved in the recruitment process or other aspects of the study.

### Evaluation and follow-up

The evaluation process will be conducted by two physiotherapists with experience in the evaluation procedures and blinded to the allocation of the participants to the different groups. Evaluations will be conducted in the following manner:

- Pre-treatment evaluation
- Evaluation immediately following a single intervention session
- Post-treatment evaluation
- Evaluation 1 month after the end of treatment
- Evaluation 3 months after the end of treatment

Evaluations will be conducted on three non-consecutive days with a maximum period of 1.5 hours per day.

### Three-dimensional analysis of upper-arm movement kinematics

**Fig. 2:**
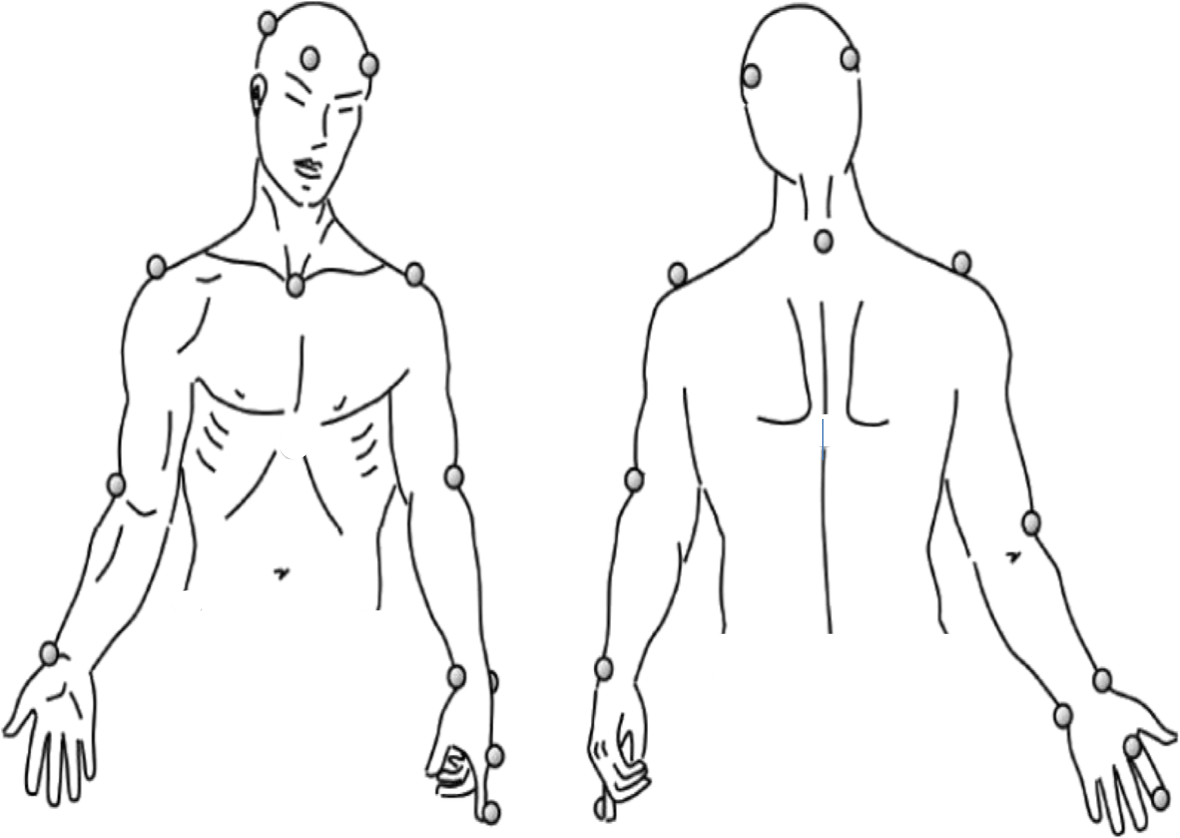
Placement of markers for three-dimensional analysis (SMARTup protocol). Schematic diagram of frontal and rear views of marker set used for 3D kinematic analysis adapted by Menegoni, 2009 [34]

Upper-arm movement kinematics will be evaluated using the SMART-D 140® system (BTS Engineering, Milan, Italy), which involves eight cameras sensitive to infrared light with a sampling frequency of 100 Hz and a synchronized video system. Passive markers will be positioned on anatomic reference points following the SMARTup protocol (Fig. 2). The markers will be attached directly to the skin using a specific adhesive tape [32, 33]. A total of 18 markers (diameter: 15 mm) will be used to identify the position of the head, trunk, and upper limb (upper arm, forearm, and hand).

The positioning of the upper limb will be reconstructed using markers. Twelve markers will be positioned bilaterally in the upper limbs: acromion, lateral epicondyle of the humerus, styloid process of the ulna and radius, head of the second metacarpus and nail of the index finger. The head position will be reconstructed using four markers: two symmetrically in the frontal bone near the timeline and two symmetrically at the back of the head. The trunk position will be estimated using markers positioned on the C7 spinous process and in the jugular notch. An additional marker will be positioned on the target of the movement trajectory.

**Fig. 3:**
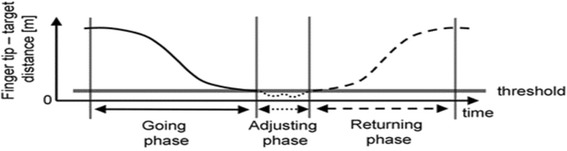
Sequential phases. Schematic representation of the distance profile between finger and target, during pointing movement. Using a threshold on the distance profile, the adjusting phase was defined Menegoni, 2009 [34]

Based on data published by Menegoni [34], the movement sequence will be in three phases: going phase (i.e., phase toward the target); adjusting phase (i.e., phase dedicated to precisely locating the target); and returning phase (i.e., phase toward initial position) (Fig. 3). At least six reach movement sequences will be performed to obtain three adequate cycles for data processing. The biomechanical model, data filtering and processing of the variables will be performed using the *SMART Analyser* software program (BTS Engineering, Milan, Italy). To evaluate changes after the intervention, the following variables will be identified in each session and the mean of the results will be calculated [32, 33, 35].

#### Total movement duration

This is calculated as the total time required for completing each task. The duration of the three phases (going, adjusting, and returning) will also be computed.

#### Mean movement velocity in the going phase

This is the mean velocity of the marker positioned on the index finger.

#### Adjusting sway index

This is the length of the path described by the fingernail in the adjusting phase; it is a measure of the adjustments made to reach the final position and represents the degree of precision.

#### Index of curvature

This is the ratio of the length of the fingernail path to the linear distance between the initial and the final pointing position; representative of movement smoothness during the going phase.

#### Average jerk

This is the mean value of the acceleration derivative (jerk) according to the equation:

$$ \mathrm{Average}\ \mathrm{jerk}=\frac{1}{T}{{\displaystyle {\int}_0^T\left[{\left(\frac{{\operatorname{d}}^3x}{\operatorname{d}{t}^3}\right)}^2+{\left(\frac{{\operatorname{d}}^3y}{\operatorname{d}{t}^3}\right)}^2+{\left(\frac{{\operatorname{d}}^3Z}{\operatorname{d}{t}^3}\right)}^2\right]}}^{1/2}\operatorname{d}t $$

in which *x*(*t*), *y*(*t*) and *z*(*t*) are the *x*, *y* and *z* coordinates of the fingernail and *T* is the movement duration. It has been shown that the average jerk index decreases with the increase in the smoothness of movement. This index is often used as a measure of the quality of selective motor control [36, 37].

#### Range of motion of the elbow and shoulder

This is calculated as the difference between the maximum and minimum values of the angle between the frontal plane (shoulder) and sagittal plane (elbow and shoulder) during the going phase [32, 33, 35]

### Electromyographic analysis

Electromyography is the most widely used assessment tool for the study of muscle activation during exercise and the intensity of contractions [38]. The electrical activity of the biceps brachii and triceps brachii muscles will be collected using the eight-camera FREEEMG® electromyography system (BTS Engineering), with a bioelectric signal amplifier, wireless transmission, and bipolar electrodes with a total gain of 2000 and a sampling frequency in the 20–450 Hz range. Impedance and the common rejection mode ratio of the equipment are >10^15^ Ω//0.2 pF and 60/10 Hz 92 dB, respectively. The motor point of the muscles will be identified and the skin will be cleaned with 70 % ethanol to minimize bioimpedance, following the recommendations of SENIAM 8 [39]. Electromyography data will be collected and digitized at 1000 frames per second using the BTS MYOLAB® software program. Electromyography data will be collected concomitantly to the collection of the kinematic data and both sets of data will be managed using the BTS® system and SMART Capture® software program.

### Quality of Upper Extremity Skills Test

This test will be used for the evaluation of manual function. It was developed to evaluate the movement patterns of normal development, which are the basis for the determination of functional upper limb performance. Evaluation of upper limb skills is divided into four areas: dissociated movements (19 items), grasp (6 items), weight bearing (5 items), and protective extension (3 items) [40]. Participants will wear a short-sleeve shirt and will not use any type of brace or adaptation during the evaluation. The rater will either demonstrate the movements or stimulate the participant orally or through the use of toys, but the participant will perform the activities without physical assistance and will maintain the position required for at least 2 s. The QUEST 40 manual furnishes specific information for each dimension and its respective scoring.

### Evaluation of upper limb spasticity

The Modified Ashworth Scale [41] will be used to evaluate spasticity. This scale quantifies resistance during the passive movement of a limb along a given range of motion and furnishes a score ranging from 0 (no increase in muscle tone) to 4 (stiffness).

Elbow extension and flexion will be analyzed. The scale will be administered by physiotherapists blinded to the allocation of the participants to the different groups. The Biomedical Technology Laboratory of the Polytechnic University of Milan has developed a portable device to measure the angle: momentum ratio to allow a high degree of precision in the evaluation of hypertonia. This device consists of two inclinometers and a strain gauge momentum sensor [42], which respectively measure the angle and momentum during elbow flexion and extension, allowing the non-invasive estimation of the passive elastic properties of the elbow. These properties are determined through the measure of the momentum of elbow flexion and extension in relation to the angle of joint motion. The momentum: angle measure will be performed throughout the entire range of motion of the elbow on the sagittal plane. Prior to the measure, the examiner will explain the procedures and how the device functions to the participants and guardians. The estimated execution time for this evaluation is approximately 15 min. The data will be stored and processed to obtain elbow angles during the movement.

### Intervention

#### Transcranial direct current stimulation

Transcranial direct current stimulation will be administered using a DC-STIMULATOR (NeuroConn, Germany) with two sponge (non-metallic) electrodes measuring 25 × 25 cm^2^ moistened in saline solution. The participants will be randomly allocated to active or sham transcranial direct current stimulation over the primary motor cortex following the procedures described by Fregni et al. [17]. The anodal electrode will be positioned over C3/C4 (international 10-20 system of electrode placement), corresponding to the primary motor cortex. The cathode will be positioned in the supraorbital region contralateral to the anode. The anode will be positioned over the primary motor cortex of the contralateral hemisphere to motor impairment. A current of 1 mA [43] will be administered for 20 min in each session. The device has a knob that allows the operator to control the intensity. Stimulation will be gradually increased to 1 mA in the first 10 s and will be gradually diminished in the last 10 s of the session. For sham stimulation, the electrodes will be placed in the same positions and the stimulator will be switched on for 30 s to give the participants the initial sensation, but no stimulation will be administered during the rest of the session. This is a valid control procedure in studies involving transcranial direct current stimulation.

### Immediate effect of transcranial direct current stimulation

Cross-sectional analysis will be conducted to determine the immediate effects of transcranial direct current stimulation. For this, the participants will be submitted to a single 20 min session of transcranial direct current stimulation over the primary cortex while at rest one month prior to the onset of the main intervention. The participants will first be instructed regarding the procedure and will remain at rest for 20 min with the electrodes in place (as described in the previous item). The evaluations will involve QUEST, three-dimensional movement analysis, surface electromyography of the biceps and triceps brachii muscles and the measurement of spasticity of the paretic upper limb. A first evaluation will be made after 20 min of rest and a second evaluation will be made after 20 min of transcranial direct current stimulation (active or sham). The raters will be blinded to the allocation of the participants to the different groups.

### Protocol for functional training of paretic upper limb

**Fig. 4:**
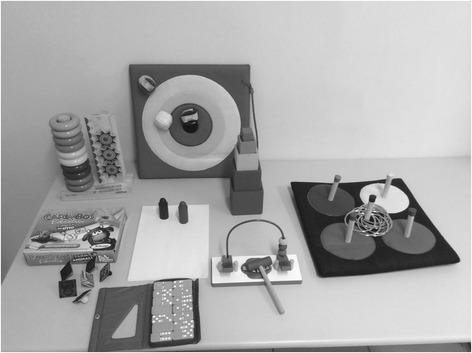
Educational toys

The therapy will be based on manual reach with the induced constraint of the non-paretic limb during the transcranial direct current stimulation session following the protocol described by Hoare et al. [6]. In each 20 min session, the participant will be seated in a chair at a height that allows 90° flexion of the hips and knees with the feet supported on the floor. A table with an adjustable height will be positioned in front of the participant. The participant will be positioned to allow free movement of the paretic upper limb for reaching and grasping and allow the visual tracking of the movement. The physiotherapist will be positioned in front of the participant to direct the movement, offering verbal encouragement and any necessary physical assistance to complete the task. Prior to the session, a variety of educational toys and objects, carefully selected for use in the task, will be placed in the therapy room (Fig. 4). To enhance their motivation and attention, participants will be asked to select the toy or object to be used during the task. The three motor strategies that will be trained are grasping, moving objects, and manual range activities. To ensure the intensive use of the paretic upper limb during the session, the non-paretic upper limb will be constrained with the use of a comfortable neoprene glove that does not allow palm grip.

After the transcranial direct current stimulation intervention protocol and until the final evaluation (three-month follow-up), the patients will receive physical therapy in two 1 hour sessions per week, directed at the functional activities of gait as well as passive stretching of the trunk, elbow, and wrist of the paretic upper limb to maintain the ranges of motion. No training or unilateral or bilateral stimulation of the upper limbs will be performed, to avoid any conflicts in the expected results of the study.

### Statistical analysis

Statistical analysis will be performed using intention-to-treat analysis. If data losses occur during the study, ‘last observation carried forward’ analysis will be employed to adjust the missing data in the follow-up evaluations. The data will be expressed as mean and standard deviation values. The two intervention groups will be analyzed for differences in anthropometric characteristics as well as clinical and functional variables using the chi-square test for categorical variables and the *t* test for continuous variables. Analysis of covariance (ANCOVA) will be employed to evaluate the effects of transcranial direct current stimulation on the outcome variables of upper limb function. For all analyses, the fixed independent variables will be group (active and sham transcranial direct current stimulation) and evaluation (before the intervention, after the intervention, at 1 month follow-up, and at 3 month follow-up). The dependent variables at baseline will be considered covariables: QUEST, outcomes obtained from three-dimensional movement analysis, electromyography, and spasticity. The ANCOVA will be used to compare effects after the interventions (active versus sham transcranial direct current stimulation), adjusting individual performance prior to transcranial direct current stimulation. This model was considered to be an advantageous approach for statistical analysis considering the nature of the study (randomized with a relatively small sample). Cohen’s *d* (effect size) will be calculated based on the difference in values between baseline and the post-intervention evaluation, with the comparison of the groups. Statistical significance will be considered for *P* < 0.05. Data will be organized and tabulated using the Statistical Package for the Social Sciences (SPSS, v.19.0).

## Discussion

This paper presents a detailed description of a prospective, randomized, controlled, double-blind, clinical trial designed to demonstrate the effects of combining transcranial direct current stimulation over the primary motor cortex and functional training of the paretic limb in children with cerebral palsy classified at levels I, II, or III of the Manual Ability Classification System. The results will be published and evidence found may contribute to the use of transcranial stimulation for this population.

This study protocol used defining standard protocol items for clinical trials. SPIRIT 2013 Statement [44].

## Trial status

Patients are being recruited at the time of submission.

## Abbreviations

ANCOVA, analysis of covariance; CONSORT, Consolidated Standards of Reporting Trials; QUEST, Quality of Upper Extremity Skills Test; ReBEC, Brazilian Registry of Clinical Trials; SPSS, Statistical Package for the Social Sciences
